# Farmers’ Work-Life Quality and Entrepreneurship Will in China

**DOI:** 10.3389/fpsyg.2019.00787

**Published:** 2019-04-30

**Authors:** Fan-Zhu Kong, Lily Zhao, Xiao-Bing Zhang, Cheng-Hung Tsai, David D. Lin

**Affiliations:** ^1^Department of Business Administration, Huaiyin Institute of Technology, Huai’an, China; ^2^Faculty of Architecture and Civil Engineering, Huaiyin Institute of Technology, Huai’an, China; ^3^Department of Business Administration, Cheng Shiu University, Kaohsiung, Taiwan; ^4^Doctoral Program, I-Shou University, Kaohsiung, Taiwan

**Keywords:** work-life quality, farmers, entrepreneurship will, China, positive psychology

## Abstract

New-generation farmers have become a vital force for entrepreneurship in their hometowns. To better promote farmers to start businesses, it’s important to know about their quality of work life. Based on a survey of the quality of work life and entrepreneurship will of farmers from three cities and eight counties in Shandong province of China, this paper analyzed the effects of farmers’ quality of work life on their entrepreneurship will in their hometowns using a Logistic regression model. Our findings show that farmers have a relatively low cognition level of their quality of work life, and their interpersonal relationship, work characteristics, material security, and family demands have significant effects on their entrepreneurship will. According to the findings, this paper proposed some suggestions for promoting farmers’ entrepreneurship will in their hometowns from the perspectives of organizational management, extrinsic entrepreneurship stimulus, and internal demand.

## Introduction

Mass entrepreneurship has become a medium- and long-term development strategy in China (Ni and Lu, 2016), and farmers are unquestionably an important component of mass entrepreneurship. According to the statistics, China has 282 million farmers by the end of 2016, a figure occupying 31.6% of the total working-age population and continuing to rise slowly. As the number of farmers goes up, Chinese governments at various levels have issued some policies to encourage and support farmers to return home to start their business. In 2015, the State Council of China issued Opinions on Supporting Farmers to Return Home to Start their Business with an aim to establishing a diversified entrepreneurship atmosphere. A prosperous landscape of farmers returning home to start their business shows up, but the number of farmers who really start their business is not optimistic. According to statistics issued by the Ministry of Agriculture of China, the number of farmers returning home to start their business totals 4.5 million, occupying about 2% of the total number of farmers in the end of 2015. This figure is far lower than the percentage (70.3%) of people believing entrepreneurship is a good career choice to the general public, also lower than the that of college students (1%) starting their business and employees in urban companies (1.2%). Such phenomenon shows that there is a big gap between farmers’ cognition of entrepreneurship and their actual practice in entrepreneurship. Thus, how to narrow such gap becomes especially important for the promotion of farmers to return home to start their businesses.

Much of existing literature on farmers’ entrepreneurship focuses on the macro-level issues (Collins, 2003; Wu and Zhang, 2012; Peroni et al., 2016). Existing research and practice show that knowledge, skill, and capital do not necessarily stimulate entrepreneurial behavior, whereas strong entrepreneurship intention is a precondition for the potential entrepreneurship behavior. Entrepreneurship is in nature a career path to realize one’s life objective, and an individual’s current living status influences its future work motivation and career development. In other words, farmers’ current living quality might be an important factor influencing their motivation for future entrepreneurship – an issue that is seldom touched on in existing literature. Hence, this paper investigates farmers’ cognition of their present quality of work life and analyzes its effect on farmers’ wills of returning home to start their businesses to identify the key factors influencing farmers’ entrepreneurship and promote the development of farmers’ entrepreneurship.

## Theoretical Basis

### Connotation of Quality of Work Life

Quality of work life, a cognitive concept, was often observed or investigated in existing literature, subject to researchers’ experience and the respondents’ personal cognition. Consequently, there is no unified definition of quality of work life. Quality of work life refers to employees’ perception and experience of their working environment which involves employees’ perception of physical and psychological well-being obtained from their work (Magid, 1994; Cascio, 1998). The definition of quality of work life that was proposed by Kirkman goes beyond the work itself and covers employees’ work, family, and leisure (Kirkan, 1989). In other words, quality of work life means employees have the right to influence decisions and improve their own work through an open communication channel adaptable to the organizational objective, thus leading to a sense of more participation, higher satisfaction, and less spiritual depression. Based on the above definitions, quality of work life can be defined in narrow and broad senses. In a narrow sense, it refers to the quality of the work itself. In a broad sense, it includes interpersonal relationship, management system, work-family relation, and concerns about employees’ balanced relationship with the organization, work, and family. It should be noted that despite the different definitions, the ultimate objective of quality of work life is to improve employees’ performance and satisfy employees’ personal demands through their perception of the work environment, management system, and interpersonal relation, and such definition is adopted by this paper. This paper will evaluate farmers’ quality of work life in a broad sense.

### Research on the Quality of Work Life of Farmers’

Research on quality of work life in western countries started in the 1930s. Chinese scholars started to study quality of work life of teachers and firm employees in the 1990s. Toward the 21st century, as default in the payment to farmers happens frequently. Up to present, farmers have become an important component of the urban labor force. Thus, the quality of work life of farmers has received increased concerns. An increasing number of Chinese scholars begin to analyze the status quo of quality of work life of farmers and propose corresponding measures to improve the quality of work life of farmers. Research shows that nearly two thirds of farmers are not satisfied with their present quality of work life which is significantly affected by factors such as their marriage status, incomes, residing location, health, family structure, and age (Jiao et al., 2008; Xu, 2016). To improve farmers’ quality of work life, the government should perfect relative laws and policies to sweep away the institutional obstacles to farmers becoming urban residents, and firms should innovate their management systems to provide equal opportunities for farmers (RaoHuixia, 2012). Some other scholars explored the potential relationship between farmers’ quality of work life and their future behaviors (Bravo-Olivas et al., 2015; Gosetti, 2017).

### Research on Entrepreneurship of Farmers

Research on entrepreneurship of farmers started in the 1990s when many farmers returned to their hometowns. Since then, Chinese scholars have conducted much fruitful research on farmers returning home to start their businesses. Such research focused on farmers’ individual characteristics, constraining factors, and policy environment. Farmers having school-age children parents in need of caring are more likely to return home to start their businesses (Liu and Song, 2002). Young male farmers who are fairly well educated and have keen insights of the market demands constitute the major entrepreneurship force (Shi et al., 2010). Besides, factors such as working experience, working years, technical skills, marriage status, family financial situations, and cognition of risks are important individual factors affecting farmers returning home to start businesses. In reality, local economic development levels, financing difficulty, entrepreneurship education, and poor training on entrepreneurship are all factors constraining farmers to return home to start businesses (Hu, 2010; Liu and Lai, 2016). A perfect entrepreneurship environment is essential for farmers to return home to start businesses. At present, there are many environmental constraints for farmers to return home to start businesses. To facilitate farmers to return home to start businesses, it’s important to create a good policy environment to provide policy support for them (Liang and Li, 2013). Meanwhile, efforts should be made to make policy information available and carry out the policies issued in an effective manner and to improve the policy service quality so as to guide and support farmers’ entrepreneurship will (Hou and Ding, 2016).

## Investigated Respondents and Data Analysis

### Investigated Respondents and Data Sources

Shandong, an agriculture province of China, has 24.2 million farmers, accounting for about 10% of the total farmers in China. In terms of geographical distribution, Shandong farmers are not evenly distributed. Farmers are densely populated in eastern Shandong while sparsely inhabited in western Shandong. To make our research findings more telling, we selected farmers living in both eastern and western Shandong. We investigated entrepreneurship will and cognition of present quality of work life of farmers returning home to start businesses through interviews and questionnaires in January through March of 2017. We sent 560 questionnaires with 496 recycled. After deleting the ineffective questionnaires, we finally obtained 467 ones, accounting for 83.4% of the total questionnaires sent.

GEM2016-17 report shows that young people are the major entrepreneurship groups in China. Thus, our investigation focused on young farmers who are between 18 and 35 years old, representing 78.2% of the total respondents and the remaining 21.8% are over 35 years old. Among all of the respondents, male farmers account for 62.6%, married farmers including divorced and widowed for 72.6%, and farmers having finished their junior middle schools for 52.4% while those having finished their senior middle schools for 21.8%. Moreover, to have a better understanding of their entrepreneurship wills, we made a deep interview of those framer workers having entrepreneurship wills in their hometowns.

The ethic regulation of conducting human subject research is at a premature stage in the researched economy. Thus, the researchers were not asked to send the research detail to a specific institution (IRB) for ethic approval, nor was is asked to gain written informed consent from questionnaire raters. This study has exempted from ethical approval in accordance to national guidelines. However, we have done every possible effort to maintain justice and rights of the researched subjects.

### Variable Selecting and Analysis Methods

International scholars usually adopt the binary variables (Yes/No that were assigned 1/0 as scores in this paper) to measure entrepreneurship will (Choo and Wong, 2006). Scholars have different understanding of quality of work life, and measuring indexes are also differentiated. However, in a broad sense, quality of work life generally involves working environment, job content, organizational management, and relationship between work and life. Quality of work life is composed of job dimension, organizational dimension, and social family and psychological dimension (Bravo-Olivas et al., 2015; Gosetti, 2017). Based on existing literature, it’s feasible to measure quality of work life from the perspectives of job, organization, and family. Hence, we selected colleague relationship, superior relationship, working environment, job content, job incomes, spare-time life, and family demands as the measuring variables, each of which consists of three questions. The respondents are asked to assign a score to each question, and the average of the scores on the three questions forms the variable score, representing farmers’ quality of work life level. Scores 1 through 5, respectively stand for very unsatisfactory, unsatisfactory, having no opinions, satisfactory, very satisfactory.

This paper aims to analyze the status quo of farmers’ quality of work life. What’s more important, it attempts to explore the relationship between farmers’ quality of work life and their entrepreneurship will. To test the relationship, we constructed a regression model in which farmers’ entrepreneurship will is expressed as the dependent variable while quality of work life as the explanatory variable. As we adopted Yes and No to measure farmers’ entrepreneurship will, we here selected the Logistic regression model, expressed as follows:

(1) $$P_{i}=F(Z_{i})=f(\alpha +\beta X_{i}+\delta )=\frac{1}{1+e^{\mathrm{zi}}}=\frac{1}{1+e^{-(\alpha +\beta X_{i})}}$$

(2) $$\ln\frac{1}{1-P_{i}}=\alpha +\beta X_{i}+\delta$$

In the above formula, *P_i_* stands for the probability of farmers’ entrepreneurship will, *F*(*Z_i_*) for the estimate function, α for the constant term of the estimate function, *X_i_* (*i* = 1,2, …,7) for the *i*th explanatory variable, β for the coefficient to be estimated of the explanatory variable, and δ for the error term.

## Empirical Analysis

### Analysis of the Status Quo of New-Generation Farmers’ Quality of Work Life

#### General Situations of New-Generation Farmers’ Quality of Work Life

Table 1 shows the scores for the seven variables of farmers’ quality of work life. Colleague relationship got the highest score above the average satisfactory level, a finding probably due to the fact that most of farmers’ colleagues are relatives and fellow-villagers, so they trust each other and have no conflicts of interest. Working environment and job content got scores between unsatisfactory and satisfactory levels, probably because farmers are usually engaged in mechanical routine jobs with repetitive job contents and long working time. Scores for superior relationship, job incomes, spare-time life, and family demands are between 3 and 4 at the satisfactory level with much room to be improved. In general, the average score for farmers’ quality of work life is 3.18 at the having no opinion level, indicating a dilemma state.

**Table 1:** Farmers’ quality of work life level.

Variable	Colleague relationship	Superior relationship	Working environment	Job content	Job incomes	Spare-time life	Family demands
**Score**	4.12	3.22	2.67	2.44	3.25	3.02	3.53

#### Analysis of the Difference of Quality of Work Life Among Different Groups of New Generation Farmers

We made *T*-test and *F*-test to find if there are demographic differences among farmers’ cognition of quality of work life. Our findings are shown in Table 2.

**Table 2:** Differences of farmers’ quality of work life and demographic variables.

Variables Demographic variable	Colleague relationship	Superior relationship	Working environment	Job content	Job incomes	Spare-time life	Family demands
Gender	*T*-test^∗^	*T*-test^∗^	*T*-test	*T*-test^∗^	*T*-test^∗^	*T*-test^∗^	*T*-test^∗^
Male	4.35	3.28	2.62	2.56	2.89	2.79	3.46
Female	3.89	3.16	2.72	2.12	3.61	3.25	3.6

Age	(1.89)	(3.01)^∗^	(4.38)^∗∗^	(4.32)^∗∗^	(5.12)^∗∗∗^	(4.46)^∗∗^	(4.06)^∗∗^
18–25	4.08	3.01	2.13	2.01	3.14	3.52	3.62
26–35	4.11	3.26	2.31	2.12	2.89	3.23	2.86
Above 35	4.17	2.31	3.39	3.57	3.19	3.72	4.11

Marriage status	*T*-test	*T*-test ^∗^	*T*-test^∗^	*T*-test^∗^	*T*-test^∗^	*T*-test^∗^	*T*-test^∗^
Married	4.14	3.31	2.75	2.51	2.95	2.76	2.88
Single	4.10	3.13	2.59	2.37	3.55	3.28	4.18

Educational	(2.21)	(2.32)	(7.38)^∗∗∗^	(8.24)^∗∗∗^	(4.26)^∗^	(6.11)^∗∗^	(4.06)^∗^
<junior school	4.11	3.11	3.12	3.01	3.33	3.30	3.62
Junior school	4.12	3.32	2.99	2.89	3.21	2.98	3.51
Senior school	4.14	3.20	2.44	2.42	3.22	2.96	3.54
>senior school	4.11	3.25	2.13	1.44	3.24	2.84	3.45

*^∗^Represents that the inter-group difference reaches the significance level of 0.05; the value within the bracket is F-test value, ^∗∗∗^represents that p < 0.001, ^∗∗^represents that p < 0.01, and ^∗^represents that p < 0.05.*

It follows from Table 2 that gender affects farmers’ cognition of their quality of work life. Except working environment, other six variables’ differences reach the significance level. In terms of colleague relationship, superior relationship, and working environment, male farmers got higher scores than female farmers, suggesting that male farmers are better at dealing with interpersonal relations and more adaptive to the environment than female farmers, a finding consistent with existing conclusions. In terms of job incomes, spare-time life, and family demands, female farmers got higher scores than male farmers, a finding possibly caused by traditional Chinese local culture that husband is responsible for earning money to support the family while wife mainly does housework and takes care of children.

Age affects farmers’ cognition of their quality of work life. Except colleague relationship, other six variables’ inter-group differences reach the significance level. The older, the better farmers know about superior relationship, working environment, and job content, indicating that farmers come to be used to the environment where they work though reluctantly over time. In terms of job incomes and family demands, farmers 26 through 35 years old have the lowest cognition level, because most of them were married and need more material incomes and time to take care of their children and family. Farmers’ cognition of spare-time life declines as they grow older, a finding possibly due to the fact that they are in nature lively and active.

Marriage status is also an important factor affecting farmers’ cognition of their quality of work life. Married farmers got higher scores than those unmarried in colleague relationship, but the difference do not reach the significance level. The difference in other six variables reaches the significance level. Married farmers got higher scores in superior relationship, working environment, and job content but lower scores in job incomes, spare-time life, and family demands than those unmarried, a finding possibly due to the fact that married farmers have rich life experiences, so they can analyze and understand what they have seen, heard, and met in a rational manner; meanwhile they need more money to meet their family demands, so they have to work hard at the expense of their spare time.

Educational background is another important factor affecting farmers’ cognition of their quality of work life. Except colleague relationship and superior relationship, other five variables reach the significance level. Those farmers below junior middle school have the highest cognition level of their quality of work life. Especially in working environment and job content, the higher the educational background, the lower the cognition level, possibly because farmers with a lower educational background have lower expectations and are thus better to be satisfied than those with a higher educational background. What’s worthwhile to be noted, farmers below junior middle school have the highest cognition level of spare-time life, whereas those above senior middle school have the lowest cognition level, and those with junior-middle-school and senior-middle-school backgrounds are just the above two groups. According to the interview results, farmers, male in particular, below senior middle school have lower needs of spare-time life, and they mainly center on daily life such as fellow villager gathering and entertainment such as playing cards or mahjong, whereas those with higher educational backgrounds have higher spiritual needs which are difficult to be met under heavy working pressure, thus their cognition level of the present state is lowered.

### Analysis of the Effects of New-Generation Farmers’ Cognition of Quality of Work Life on Their Entrepreneurship Will

To test the effects of new-generation farmers’ cognition of quality of work life on their entrepreneurship will, we made a binary regression analysis adopting SPSS20.0 according to the above formula. According to prior conclusions, the demographic variable may affect farmers’ entrepreneurship will, so it was taken as a control variable to prevent deviation of the research findings during the regression analysis. Values of Cox & Snell *R*^2^ and Negelkerke *R*^2^ are respectively 0.379 and 0.413, the fitting effect being good as shown in Table 3.

The regression results show that farmers’ cognition of quality of work life significantly affects their entrepreneurship will. Specifically speaking, the regression coefficient of colleague relationship is 5% and positive, indicating that colleague relationship has a positive correlation to entrepreneurship will, i.e., the better the colleague relationship, the stronger the entrepreneurship will. Our interview results also demonstrate that part of the respondents’ entrepreneurship will positively be affected by their colleagues and fellow villagers, and farmers with similar entrepreneurship wills are closer to each other trust each other more than those without similar entrepreneurship wills do. The regression coefficient of superior relationship is also 5% but negative, denoting that superior relationship has a negative correlation to entrepreneurship will, i.e., the poorer the farmers’ relationship with their superiors, the stronger the farmers’ entrepreneurship will in their hometowns. Our interview results show that poor superior relationship is not the major reason but a blasting fuse for farmers’ entrepreneurship will, as they think they will also return home to start their businesses even if they have a good relationship with their superiors.

**Table 3:** Regression model of Entrepreneurship will of farmers returning home to start businesses.

Independent variables	Regression coefficient	S.E.	Wald	Significance level
Colleague relationship	0.325^∗∗^	0.207	6.781	0.029
Superior relationship	−0.378^∗∗^	0.217	7.016	0.032
Working environment	−0.112	0.103	0.992	0.177
Job content	−0.231^∗^	0.187	4.997	0.068
Job incomes	0.248^∗^	0.221	5.577	0.076
Spare-time life	−0.251^∗^	0.167	5.975	0.079
Family demands	−0.467^∗∗∗^	0.374	9.172	0.000
Constant item	3.152^∗∗∗^	1.971	37.267	0.000
No. of samples	467			
Nagelkerke *R*^2^	0.413			
Cox & Snell *R*^2^	0.379			
Overall Percentage	91.2%			

*^∗∗∗^, ^∗∗^, ^∗^ respectively represents that it is significant at 1, 5, and 10%.*

In terms of the job itself, the regression coefficient of job content is 10%, whereas the regression coefficient of working environment is insignificant yet negative, indicating that farmers’ being unsatisfied with the job content is one of the factors pushing them back home to start businesses and that farmers care more about job content than working environment. In recent years, the government has promulgated some policies aiming to protect farmers’ interests, so farmers’ working environments become better than ever before. On the other hand, as the national free education becomes popular, farmers are better educated and have greater demands correspondingly. Meanwhile, as the government invests more in agriculture, rural areas, and farmers, farmers’ incomes have increased dramatically. Thus, most of farmers do not consider job incomes as their first demand any more. Instead, they care more about the job significance and their own interests.

Entrepreneurship needs capital inputs. Lack of capital is an obstacle to farmers’ starting businesses, so better job incomes may push farmers back home to start their businesses. The regression results show that the regression coefficient of job incomes is 10% and positive, indicating that the better the job incomes, the stronger the farmers’ entrepreneurship will. Further interview shows that better job incomes help to resolve farmers’ problem of capital shortage in the early stage of their entrepreneurship as well as relieve their family financial difficulties once they fail in entrepreneurship. Part of the respondents maintain that starting businesses in hometown is not only a personal issue but also a behavior which can bring glory on their ancestors and improve their family’s position and reputation in their hometowns. The regression coefficient of spare-time life is also 10% but negative, indicating that spare-time life has a negative correlation to entrepreneurship will, possibly because rich spare-time life needs steady incomes, but entrepreneurship faces great uncertainties.

The regression coefficient of family demands is 1% but negative, suggesting that family demands have the greatest effect on farmers’ entrepreneurship will. Family demands here mean educating children and taking care of old parents, so farmers having children left at home and old parents waiting to care about are more likely to return home to start businesses. In general, entrepreneurship needs energy and time inputs. Even if farmers starting businesses in their hometown do not have enough time to take care of their family, they can meet their family members’ emotional demands in case of emergency, and this might be the most important reason for farmers to return home to start businesses.

## Conclusion and Suggestions

At present, young adult farmers have become the major force of returning-home entrepreneurship. The results presented in this study is a good reference for the steady development of the rural areas. In general, the major motivation for young adult farmers to return home and start businesses stemmed from external stimulus and internal demands. To encourage farmers to return home to start businesses, efforts should be made to stimulate their entrepreneurship will and enthusiasm and improve their quality of work life.

Furthermore, farmers’ starting new ventures should constantly optimize their organizational management and improve the interpersonal relationship. A harmonious interpersonal relationship within the organization helps to improve farmers’ commitments to the organization, better understand their occupational expectations, get ready for possible vacant positions in advance, and reduce the potential resultant organizational losses. Our empirical analysis shows that farmers have a low cognition of the superior relationship, which in turn affects their quality of work life. Thus, managers should constantly optimize the internal organizational management systems and create harmonious interpersonal relations to enhance the overall organizational performance and facilitate potential entrepreneurs to return home to start businesses.

As for entrepreneurship stimulus, the government should take practical measures to stimulate and support farmers to start businesses at their hometown. Besides, the government should also provide information, technical training, and policy guidance on entrepreneurship for farmers to prevent them from starting businesses in an aimless way. Moreover, the government should also endeavor to perfect policies on capital support, favorable taxation, and medical care to improve farmers’ entrepreneurship expectations and in turn intensify their entrepreneurship will.

As for internal entrepreneurship demands, efforts should be made to create a multi-level educational and training system to stimulate farmers’ internal entrepreneurship will. Our findings show that current farmers have a low cognition level of entrepreneurship and active entrepreneurship based on internal motive is rare. Thus, efforts should be made to create a multi-level educational and training system to improve farmers’ cognition level of entrepreneurship and inspire their internal entrepreneurship will to transfer the passive idea of “I am wanted to start business” to an active idea of “I want to start business.”

## Data Availability

The datasets generated for this study are available on request to the corresponding author.

## Ethics Statement

An ethics approval was not required as per applicable institutional and national guidelines and regulations. The informed consent of the participants was implied through survey completion.

## Author Contributions

F-ZK led the project and wrote for the Introduction and Literature Review. LZ and X-BZ collected the data and conducted analysis, and worked with C-HT and DL to write up the Results, Discussions, and Conclusion sections.

## Conflict of Interest Statement

The authors declare that the research was conducted in the absence of any commercial or financial relationships that could be construed as a potential conflict of interest. The handling Editor declared a shared affiliation, though no other collaboration, with one of the authors C-HT.
